# The effect of microbial selection on the occurrence-abundance patterns of microbiomes

**DOI:** 10.1098/rsif.2021.0717

**Published:** 2022-02-09

**Authors:** Román Zapién-Campos, Michael Sieber, Arne Traulsen

**Affiliations:** Max Planck Institute for Evolutionary Biology, Plön, Germany

**Keywords:** ecology, neutral theory, niche theory, migration, biodiversity, mathematical model

## Abstract

Theoretical models are useful to investigate the drivers of community dynamics. In the simplest case of neutral models, the events of death, birth and immigration of individuals are assumed to only depend on their abundance—thus, all types share the same parameters. The community level expectations arising from these simple models and their agreement to empirical data have been discussed extensively, often suggesting that in nature, rates might indeed be neutral or their differences might not be important. However, how robust are these model predictions to type-specific rates? Also, what are the consequences at the level of types? Here, we address these questions moving from simple neutral communities to heterogeneous communities. For this, we build a model where types are differently adapted to the environment. We compute the equilibrium distribution of the abundances. Then, we look into the occurrence-abundance pattern often reported in microbial communities. We observe that large immigration and biodiversity—common in microbial systems—lead to such patterns, regardless of whether the rates are neutral or non-neutral. We conclude by discussing the implications to interpret and test empirical data.

## 1. Introduction

Theoretical models have been instrumental in understanding ecological systems. Historically, a handful of puzzling natural observations have motivated their development—from the limits of exponential growth by Malthus [1] to the competition of species by Lotka & Volterra [2,3].

The stark difference of the frequencies of species within communities is one such observation. While few species are very abundant, many others barely appear in community surveys [4]. Two hypotheses have dominated the scientific discussions. On the one hand, it is proposed that biotic interactions and environmental filtering make trophically similar species occupy different niches, which allows differences in abundance while preserving diversity. This is known as niche theory [5]. Alternatively, Hubbell *et al.* [6] have emphasized that even if niche differences are discounted, so only species’ abundances matter, random fluctuations can lead to the patterns of abundance and diversity observed in nature. This is known as neutral ecological theory [7].

Despite their stringent assumptions, neutral models often predict patterns observed in communities as different as the tropical rainforest of Barro Colorado island [7] and host-associated microbiomes [8–10]. With time, neutral models have become null hypotheses used to discard the need for complex mechanistic explanations in data at the community level [6].

However, how does a neutral model work? In a neutral model, the death and birth of individuals account for changes in community composition, but because each rate is identical for all types, after some time, stochastic drift leads to the extinction of all but one type [11]. Thus, to preserve diversity, an external source of individuals by immigration or speciation is needed. Here, neutral theory builds upon island biogeography. In this theory, MacArthur & Wilson [12] have modelled the community composition of small habitats (islands) connected by migration to a larger habitat (mainland). In neutral models, a local community commonly receives individuals from an external and larger community [13]. Such a community can itself undergo internal changes or, by separation of time scales, assumed to be constant [7,13].

Initially, neutral models have been used in macroecology to address the patterns of diversity and abundance of species [6,7]. More recently, driven by developments in sequencing technologies, the study of patterns of occurrence and mean frequency in microbial communities has become possible [14,15]. At this scale, ecological drift also seems to greatly influence the community dynamics, leading to hypothesize that many microbial taxa could be classified as neutral [10,16]. However, few taxa, referred to as non-neutral, have occurrences and frequencies different than neutrally expected. It has been suggested that the last group might include, among others, pathogens and symbionts [10].

At least two possibilities could lead to deviations from neutrality. Either different processes from those in the neutral model are necessary, or, alternatively, not all the parameters of the model are actually neutral. Both of these lead to develop models of selection [11]. Although many such models have been developed from niche theory assumptions, fewer have been developed from a neutral theory basis [6,17]. A direct connection from neutral to selective models would allow the comparing of their patterns while acknowledging that both might be operating simultaneously. Neutral models can be rejected when the patterns are observed to not conform to their predictions. However, ‘compatible with a neutral model’ does not immediately imply neutrality: non-neutral processes can only be rejected after ensuring that they cannot produce ‘neutral’ patterns in data [17].

Neutral and niche models have been connected in several ways [13,18,19]. Some authors have assumed that the rate of types are solely determined by the environment, finding that neutrality might overshadow the niche structure effect [20], depending on diversity, dispersal, and niche overlap [18]. Alternatively, using Lotka–Volterra models with immigration, the effect of competitive interactions has been studied. Early models focused on intraspecific [21] or interspecific [22] competition. Later on, both were considered simultaneously. Haegeman & Loreau tuned the niche overlap using symmetric interactions to investigate the success behind the neutral assumption [19]. Kessler & Shnerb classified the dynamics emerging from interspecific interactions, finding that the neutral case links all classes [13]. Focusing on intraspecific interactions, Gravel *et al.* studied the influence of immigration, suggesting a continuum from competitive to stochastic exclusion [18]. Throughout these studies, diversity, community size, and environmental fluctuations seem to have great relevance, as pointed out by Chisholm & Pacala [20] and Fisher & Mehta [33].

This previous research has proven useful to bridge neutral and selective theories. The link has been instrumental to consider migration, speciation, and stochastic demography key components in ecology. Along this line and motivated by the particularities of microbial communities, large community size and taxa diversity [14,16,23], here we investigate the commonly observed occurrence-abundance pattern in neutral and non-neutral contexts. Similarly to Sloan *et al.* [16] and Allouche & Kadmon [22], we model death, birth and immigration within a community, but in contrast to these neutral models, type-specific growth and death rates are determined by the environment.

### 1.1. Terminology

Within this work, we define key terms in the following way:

*biodiversity*: the total number of different types in a community;

*ecological neutrality*: the equality of death and growth rates for all individuals in a community;

*environmental filtering:* the set of environmental factors impeding the establishment of a type;

*nche:* the set of biotic and abiotic factors sustaining the existence of a type in a community;

*niche overlap:* the set of niche factors shared by multiple types which lead them to compete;

*occurrence frequency*: the chance of a type of having at least one individual per community; and

*mean frequency*: the mean relative abundance of a type across the communities.

## 2. Results

### 2.1. A spatially implicit death–birth model with immigration

We consider a set of local communities connected by immigration to a larger community which contains multiple types of individuals. While local communities change as a result of the death, birth and immigration of individuals, the larger community changes on a much longer time scale—so immigration to local communities can be assumed to be constant. To derive a dynamical equation of a local community composition, we account for the events that change the frequency *x*_*i*_ of each type *i* = 1, …, *S* within each local community. Individuals die with a rate proportional to the product *x*_*i*_ *ϕ*_*i*_ of their frequency *x*_*i*_ and their death rate *ϕ*_*i*_. Additionally, they are born proportional to the product *x*_*i*_ *f*_*i*_ of their frequency and their constant growth rate *f*_*i*_—or arrive with a fraction of the immigration rate *m* that reflects their frequency *p*_*i*_ in the external environment. Combining these processes, we obtain:

2.1 $$\frac{dx_{i}}{dt}=f_{i}x_{i}-\phi_{i}x_{i}+mp_{i}.$$

Assume for now an equal death rate for all types within a community, *ϕ*_*i*_ = *ϕ*, so only *f*_*i*_, *m* and *p*_*i*_ are free parameters. To hold the community size constant, we use $\sum_{i}\;dx_{i}/dt=0$ to find $\phi =\bar{f}+m$, where $\bar{f}=\sum_{j}x_{j}f_{j}$ is the average growth rate of a randomly selected individual. In this way:

2.2 $$\frac{dx_{i}}{dt}=x_{i}(f_{i}-\bar{f})+m(p_{i}-x_{i}).$$

Without immigration, *m* = 0, equation (2.2) shows that only types whose growth rate is larger than the average increase. After sufficient time, only the type with the largest growth rate remains. Coexistence is only possible in the neutral case, where all types have the same growth rate, $f_{i}=\bar{f}$. There, the initial frequencies remain unchanged. Immigration, *m* > 0, creates an equilibrium that resembles the external composition, *p*_*i*_, that for sufficiently large immigration might promote coexistence, especially if types with small growth rate migrate more. Similar results are obtained if we assume equal growth rate for all types, *f*_*i*_ = *f* in equation (2.1) instead. In the general case, growth and death rates have opposing effects.

Equation (2.1) provides useful insights about the dynamics and equilibria; however, only a stochastic model allows us to compute observables such as the occurrence frequency and the variance. To develop such a model, we track the vector of absolute abundances instead, **n**, and list the transition rates that change it. The increase of type *i* by one individual occurs at the expense of the decrease of type *j*:

2.3 $$R(n\to n+e_{i}-e_{j})=\phi_{j}\frac{n_{j}}{N}(f_{i}n_{i}+mp_{i}).$$

Here, **e**_*i*_ and **e**_*j*_ are vectors whose *i*th or *j*th element equals one and zero elsewhere. The fixed population size of the community is given by *N*. The master equation accounts for changes in the probability of observing the community composition **n** through time:

2.4 $$\begin{array}{cc} \frac{\partial P(n,\;t)}{\partial t}=\; & \underset{\mathrm{probability}\;\mathrm{influx}}{\underbrace{\sum_{i,j;i\neq j}P(n+e_{i}-e_{j},\;t)R(n+e_{i}-e_{j}\to n)}}\;\;-\;\\ \; & \;\underset{\mathrm{probability}\;\mathrm{outflux}}{\underbrace{\sum_{i,j;i\neq j}P(n,\;t)R(n\to n+e_{i}-e_{j}),}}\; \end{array}$$

where *P*(**n**, *t*) is the probability density of community composition **n** at time *t*.

In this work, we investigate the probability distribution at equilibrium, i.e. the state where the master equation equals zero. In this case, the influx and outflux to each state balance each other, ending up with a system of equations that can be solved to find *P*(**n**). For communities composed of two types (*S* = 2), detailed balance [24] is fulfilled and leads to a recurrence equation of the arbitrarily denoted type 1:

2.5 $$P(n_{1})=P(0)\prod_{n=(0,N)}^{\left(n_{1}-1,1\right)}\frac{R(n\to n+e_{1}-e_{2})}{R(n+e_{1}-e_{2}\to n)},$$

satisfying $\sum_{0}^{N}P(n_{1})=1$. In this case, the transition rates depend on the single variable *n*_1_, using *n*_2_ = *N* − *n*_1_ and *p*_2_ = 1 − *p*_1_.

For communities with more than two types (*S* > 2) analyses are more challenging, as all possible compositions must be considered. This is particularly true for microbial communities, where many types interact (10^1^ to 10^4^ taxa are common) in large communities (10^3^ to 10^14^ individuals). Although a recurrence equation exists [22], the exponential increase in the number of states and transitions with *S* and *N*, make its computation unfeasible. This is a problem common to microscopic and even mesoscopic descriptions, which has been termed ‘the curse of dimensionality’ [25]. In neutral models, the equality of rates allows us to reduce analyses to a single dimension—that of a focal type [16]. However, unless density dependence is neglected, non-neutral models are inherently multidimensional, as transitions depend on the current community composition [26,27]. In this case, we rely on stochastic simulations [28] to compute the mean frequency and occurrence frequency at equilibrium.

### 2.2. The neutral expectation

We start by considering the neutral case—a situation where the rates of all types are equal (*f*_*i*_ = *ϕ*_*i*_ = 1 for all *i* in {1, …, *S*}). In contrast to the deterministic model at equilibrium, equation (2.2), the frequencies of single stochastic realizations change through time, driven by the probabilistic nature of events. As a result, a distribution of frequencies centred at the value set by the source of immigrants (*p*_*i*_) emerges. The spread of this distribution inversely depends on the magnitude of the immigration, *m*.

**Figure 1.**
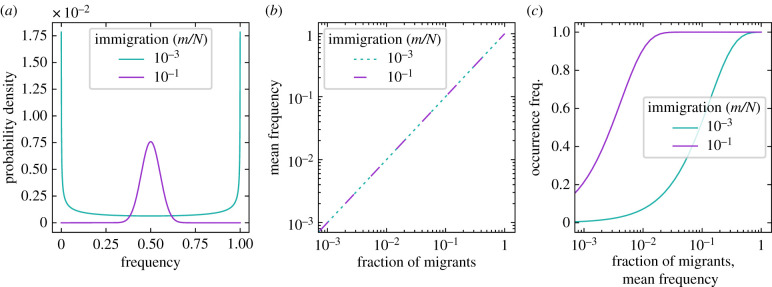
Expected equilibrium of a type if rates in the community are neutral. The frequency is the relative abundance of a type *i* across the communities, *n*_*i*_/*N*, while the mean frequency is given by *E*(*n*_*i*_)/*N*. The occurrence frequency is the chance of type *i* having at least one individual per community, *P*(*n*_*i*_ ≥ 1). (*a*) If the immigration parameter *m* is very small, the population either goes extinct or reaches fixation. A larger immigration reduces the variation in frequency, centred at its fraction of immigrants (here *p*_1_ = 0.5). (*b*) The mean frequency increases with the fraction of immigrants *p*_1_, but it is independent of the immigration rate *m*. (*c*) The occurrence frequency increases with the fraction of immigrants (*p*_1_), but in an S-shaped manner that depends on *m*. Deviations from these patterns can indicate non-neutral rates [10]. Lines show numerical solutions of the exact solution equation (2.5), with community size *N* = 10^3^.

As shown in figure 1*a*, large immigration drives the equilibrium distribution towards its mean value, *p*_*i*_. On the contrary, without or with little immigration, the distribution splits. Thus, the frequencies zero (no individuals of the *i*th type) and one (only individuals of the *i*th type) are the most probable, decaying towards intermediate frequencies. This is a consequence of noisy fluctuations that, for a single realization, lead to the extinction of all but one type. Whether the frequency one or zero is most likely depends on the proximity of the initial state.

The mean frequency of the stochastic model corresponds to the frequency of the deterministic model. As shown in figure 1*b*, regardless of the total immigration, the mean frequency of a type increases linearly with the fraction of migrants of its kind.

Besides the mean frequency, one of the simplest, but most informative observables is the occurrence frequency of individuals of a given type in the community. In other words, the probability of observing at least one individual of that type, *P*(*n*_*i*_ ≥ 1). Immigration increases this probability up to the point where the type is always observed in the community (figure 1*c*). Importantly, this probability does not increase linearly with the fraction of migrants. Instead, an S-shaped curve is observed, where changes of immigration of rare or abundant types do not modify their occurrence.

Using two simple observables, the mean frequency and the occurrence frequency, we can describe the state of types within a community. In the following, we relax the assumption of neutrality—not enforcing equal growth and death rates. Then, we compare both observables to their neutral expectation.

### 2.3. Immigration lessens the effect of growth and death differences

To understand the effect of non-neutral rates, we start from a community composed of only two types. Furthermore, we assume only a single difference in the rates, either in *f*_*i*_ or *ϕ*_*i*_.

**Figure 2.**
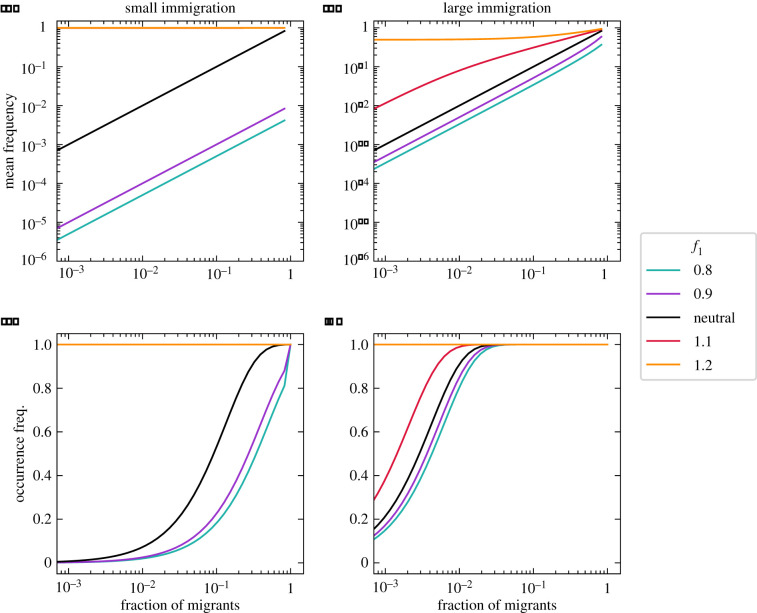
Effect of non-neutral growth rates on the equilibrium of a community with two types. The growth rate between the two types differs (*f*_1_ ≠ *f*_2_ = 1), but their death rates are equal (*ϕ*_1_ = *ϕ*_2_ = 1). In contrast to the neutral (*f*_1_ = *f*_2_ = 1) expectation, a lower growth rate of one type (*f*_1_ < *f*_2_) reduces its mean frequency and occurrence. The change can be of several orders of magnitude. Inversely, a larger growth rate of one type (*f*_1_ > *f*_2_) increases its mean frequency and occurrence. The effect of growth rate differences on the internal dynamics is most prominent for small immigration rate (*a*,*c* with *m*/*N* = 10^−3^), especially for slowly growing types. It becomes weaker with larger immigration (*b*,*d* with *m*/*N* = 10^−1^). Lines show numerical solutions of the exact solution equation (2.5), with community size *N* = 10^3^.

**Figure 3.**
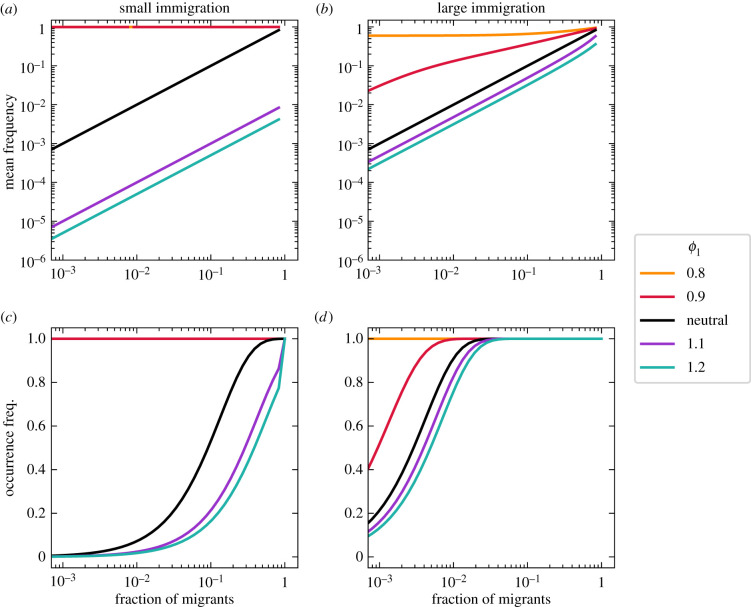
Effect of non-neutral death rates on the equilibrium of a community with two types. The two types have different death rates (*ϕ*_1_ ≠ *ϕ*_2_ = 1), but identical growth rates (*f*_1_ = *f*_2_ = 1). Differences in death rates modify the mean frequency and occurrence of both types, in particular for small immigration (*a*,*c* with *m*/*N* = 10^−3^). Larger immigration (*b*,*d* with *m*/*N* = 10^−1^) reduces differences to a scenario similar to the neutral (*ϕ*_1_ = *ϕ*_2_ = 1) expectation in a similar fashion to differences in growth rate (figure 2). Lines show numerical solutions of the exact solution equation (2.5), with community size *N* = 10^3^.

Let us start with a disadvantageous type, i.e. a growth rate below one (*f*_1_ < 1) or a death rate above one (*ϕ*_1_ > 1). The associated type has a reduced mean frequency that preserves its linear relationship to the fraction of immigrants (figure 2*a*,*b* and figure 3*a*,*b*). However, in contrast to the neutral expectation, immigration does play a role, as large migration can reduce the changes occurring in the internal community dynamics (compare panels (*a*) to (*b*) in figures 2 and 3). In this context, the neutral type (*f*_2_ = *ϕ*_2_ = 1) benefits from the reduced proliferation of its partner, thus, gaining in frequency, especially if most immigrants belong to the neutral type.

A similar picture arises for the occurrence pattern. While the non-neutral type occurs less frequently, the neutral type thrives, occurring more often than when both types are neutral (figures 2*c*,*d* and 3*c*,*d*). The change can be as severe as losing all non-neutral individuals from the community (figures 2*c* and 3*c*). Crucially, large immigration *m* can prevent this (compare panels *c* to *d* in figures 2 and 3), even if most migrants are of the neutral type.

Let us now focus on an advantageous deviation from neutrality, a non-neutral growth rate is above one (*f*_1_ > 1) or a death rate below one (*ϕ*_1_ < 1). Once the roles are reversed, the mean frequency and occurrence patterns mirror the previous results (figures 2 and 3). Although changes produced by non-neutrality in growth (*f*_1_ ≠ 1) or death (*ϕ*_1_ ≠ 1) rates are qualitatively similar, they show quantitative differences.

We conclude that even for the simplest community (one with two types), one non-neutral rate is enough to change the community occurrences and abundances substantially from their all-neutral expectation. This is more visible through the mean frequency (as changes of several orders of magnitude are possible) and for communities with little external migration—where the internal dynamics is more important.

### 2.4. Neutral and non-neutral patterns are similar at the community level but full of differences at the level of types

Communities with two types can be constructed *in vitro*. However, in nature, communities are much more diverse, especially for microbes. We have produced random instances of such diverse communities, sampling growth and death rates, *f*_*i*_ and *ϕ*_*i*_, from a normal distribution with mean one and a desired standard deviation. Similarly, we have produced random fractions of migrants, *p*_*i*_, just conditioned on the Gini index of the community [29]:

2.6 $$G=\frac{1}{S-1}\sum_{i,j}|p_{i}-p_{j}|.$$

This number that indicates the asymmetry in immigration between types from zero to one, allow us to compare communities quantitatively, regardless of their number of types *S*. As an example, for *G* = 0 the fractions of migrants are identical for each type, while for *G* = 1 the source pool only contains a single type.

**Figure 4.**
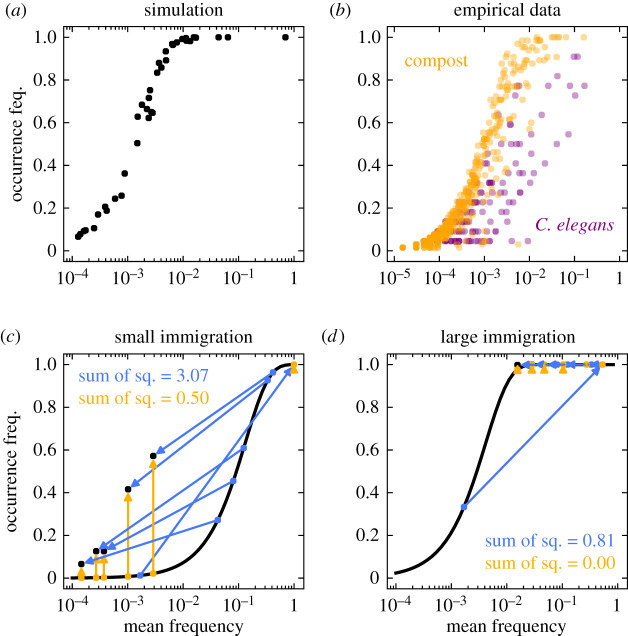
Occurrence-abundance pattern in general non-neutral communities. (*a*) The overall non-neutral pattern of a diverse community largely resembles neutral patterns at the community level, see figure 1*c*, (*b*) as well as the pattern of two representative empirical microbiomes [10]. (*c*) At the level of types, the change from neutrality can be very large (blue arrows), shown here for *m*/*N* = 10^−3^. In general, the mean frequency does not equal the fraction of immigrants *p*_*i*_. Making the assumption of equality underestimates the change from neutrality (yellow arrows). (*d*) Similar to a community with two types, figures 2 and 3, the overlap to the neutral expectation increases when immigration, *m*, is increased—here to *m*/*N* = 10^−1^. The growth and death rates, *f*_*i*_ and *ϕ*_*i*_, were sampled from a normal distribution with mean 1 and standard deviation 0.1, where *P*(*f*_*i*_ < 0.8) = *P*(*f*_*i*_ > 1.2) ≈ 0.023 and *P*(*ϕ*_*i*_ < 0.8) = *P*(*ϕ*_*i*_ > 1.2) ≈ 0.023. The fractions of migrants *p*_*i*_ range from 10^−4^ to 10^−1^ and have a *G* ≈ 0.6, equation (2.6), indicating intermediate immigration asymmetry. Except from the immigration rate *m*, all rates in (*c*,*d*) are equal. The community size is *N* = 10^3^ throughout and we simulated 10^3^ hosts. The community shown in (*a*) corresponds to figure 5*c*. We focus on the large immigration regime, *m*/*N* = 10^−1^, in figures 5 and 6.

**Figure 5.**
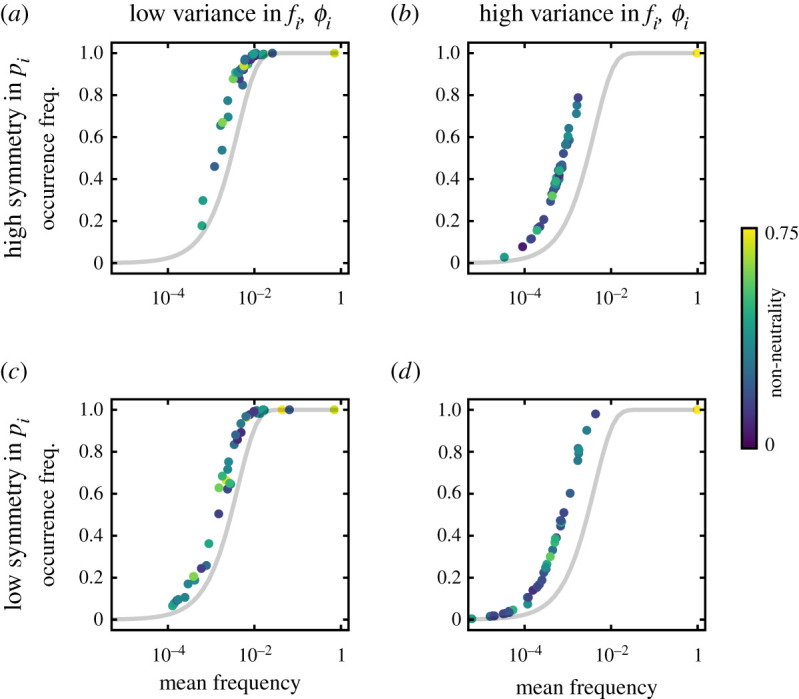
Occurrence-abundance pattern for different levels of asymmetry in the parameters. The S-shaped pattern is robust to various asymmetries in the immigration, *p*_*i*_, and growth and death rates, *f*_*i*_ and *ϕ*_*i*_. Each community has 40 types. For low symmetry in immigration, the types span the range more widely. Colours from dark to light indicate how non-neutral a type is, quantified as the euclidean distance from the neutral parameter set (*f*_*i*_, *ϕ*_*i*_) = (1, 1). Types overlap regardless of their non-neutrality. However, very non-neutral types can lead to a smaller mean frequency of the others. The fractions of immigrants, *p*_*i*_, have a *G* ≈ 0.3 (*a*,*b*) or *G* ≈ 0.6 (*c*,*d*). The growth and death rates, *f*_*i*_ and *ϕ*_*i*_, were sampled from a normal distribution with mean 1 and standard deviation 0.1 (*a*,*c*) or 0.2 (*b*,*d*). In the last case, *P*(*f*_*i*_ < 0.8) = *P*(*f*_*i*_ > 1.2) ≈ 0.159 and *P*(*ϕ*_*i*_ < 0.8) = *P*(*ϕ*_*i*_ > 1.2) ≈ 0.159. Immigration is *m*/*N* = 10^−1^, with community size *N* = 10^3^ and 10^3^ hosts were simulated.

Using these parameters, we have computed the occurrence and abundance frequency of all types in a certain community. Interestingly, the community patterns that we observe are very similar to those expected from neutrality (figure 4*a* compared to figure 1*c*)—even if asymmetries of growth, death, and immigration increase (figure 5). The theoretical patterns resemble empirical observations in host-associated and environmental microbiomes (figure 4*b*). In particular, large immigration together with high biodiversity consistently lead to these patterns (figure 5). This indicates that neither neutrality nor non-neutrality, but large immigration and biodiversity can explain these patterns. A major difference from neutrality can occur when types with a very large advantage drive the other types to smaller mean frequencies (figure 5*b*,*d*).

Even when neutral and non-neutral patterns are similar at the community level, we observe large differences at the level of types. While in the ‘all-neutral’ case, the mean frequency equals the fraction of migrants, *E*(*n*_*i*_)/*N* = *p*_*i*_, this is not the case in a non-neutral scenario (figure 4*c*,*d*). Neither is for the occurrence frequency. The distance from the neutral expectation of each type is not simply related to the level of non-neutrality of its own parameters. Rather, neutral and non-neutral types fall all along the occurrence-abundance curve (figure 5), highlighting the inherent multidimensionality determining the equilibrium of these communities.

**Figure 6.**
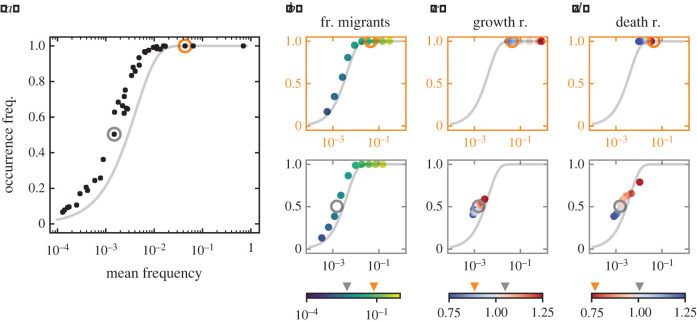
Effect of growth, death, and immigration at the level of types. (*a*) The community shown corresponds to figure 5*c*, with *G* ≈ 0.6 for *p*_*i*_, and *f*_*i*_ and *ϕ*_*i*_ drawn from N(1,0.1). Two types are spotted by circles based on their occurrence—intermediate and large occurrence. Single parameters are modified in (*b*–*d*) for both types. Arrows in the colour bars indicate their original values. (*b*) For various fractions of migrants, *p*_*i*_, non-neutral types recreate the S-shaped pattern; occurrences below one have a mean frequency smaller than neutrally expected. (*c*) Different growth rates, *f*_*i*_, only lead to local changes on occurrence and mean frequency. (*d*) Different death rates, *ϕ*_*i*_, mirror the effect of changing growth rates qualitatively, but we observe that non-neutral types can fall on, above, or below the neutral expectation. Immigration is *m*/*N* = 10^−1^, with community size *N* = 10^3^ and 10^3^ hosts were simulated.

To investigate the effect of single parameters at the level of types, we chose two representative types—one with intermediate occurrence and one with large occurrence (figure 6*a*). Our results show that types do not remain on or far from the neutral expectation. Rather, the relative magnitude of their growth and death rate, *f*_*i*_ and *ϕ*_*i*_, is crucial to observe simultaneous decrease or increase in occurrence and mean frequency (figure 6*c*,*d*). Nevertheless, in the large immigration regime, changes are local and heavily determined by the fraction of migrants (figure 6*b*).

### 2.5. The importance of niche structure

So far we have used our model to compute observables based on known parameters. However, we can invert this process to infer parameters from simulations or experimental data.

Particularly relevant is the possibility of testing niche structure in data [8–10,16]. Our model indicates care is needed to quantify the true difference from neutrality (figure 4*c*,*d*). In fact, the comparison of the selective case to the neutral case can only be inferred after fitting all parameters of the general model (*m*, *p*_*i*_, *f*_*i*_ and *ϕ*_*i*_ for all *i*). This is in contrast to the—often used—method by Sloan *et al.* for neutral conditions, where only the immigration rate *m* is fitted, while all growth and death rates are assumed *f*_*i*_ = *ϕ*_*i*_ = 1, and the fraction of migrants *p*_*i*_ equalled to the mean frequency *E*(*n*_*i*_)/*N*. Our results indicate these assumptions on the data lead to underestimate niche structure (figure 4*c*,*d*), especially in large communities with many types. Moreover, the consistent occurrence-abundance pattern that we observe (figure 5), and often reported in data [8–10], can emerge from a general death–birth processes with immigration, equation (2.3), not just from a neutral process (where *f*_*i*_ = *ϕ*_*i*_ = 1 for all *i*). Niche structure—and thus neutrality—can not be discarded or confirmed if certain parameters are fixed *a priori* [16].

The large number of parameters to be fitted requires large datasets. For a community with *S* types, 3*S* + 1 parameters must be fitted, thus usually requiring much more than 3*S* + 1 data points. The 2*S* data points obtained from the occurrence and mean frequencies are not sufficient. We propose to include additional observables that can be readily computed from data [30]. These might include, but not be limited to, the moments of the frequency. From this set of observables, available Bayesian methods [31] can be used to infer the parameters [15].

**Figure 7.**
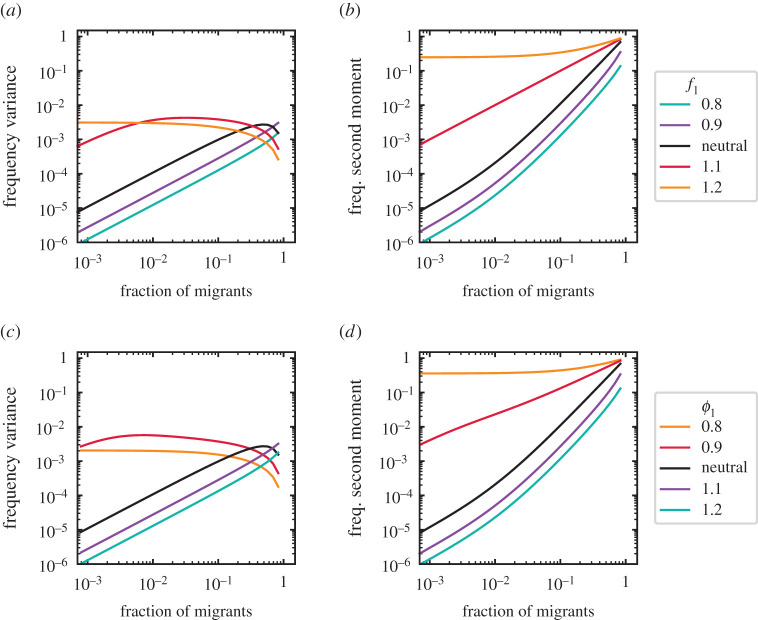
Variance and second moment of the frequency. A community with two types is considered. (*a*,*b*) One type has non-neutral growth rate (*f*_1_ ≠ *f*_2_ = 1) but neutral death rate (*ϕ*_1_ = *ϕ*_2_ = 1), or (*c*,*d*) a neutral growth rate (*f*_1_ = *f*_2_ = 1) but non-neutral death rate (*ϕ*_1_ ≠ *ϕ*_2_ =1). (*b*,*d*) The second moment, given by $\sum_{n_{1}=0}^{N}P(n_{1}/N){\left(n_{1}/N\right)}^{2}$, is above or below the neutral expectation for *f*_1_, *ϕ*_1_ > 1 and *f*_1_, *ϕ*_1_ < 1. Note how this is similar to the mean—first moment—of the frequency, figure 2*b* and figure 3*b*. This moment increases continuously with the fraction of migrants, *p*_*i*_, while the variance reaches a maximum at intermediate *p*_*i*_ (*a*,*c*). In contrast to the second moment, the variance of different growth and death rates overlaps. Differences in death rates mirror the effect of growth rate differences qualitatively. Immigration is *m*/*N* = 10^−1^, with community size *N* = 10^3^. Lines show numerical solutions of the exact solution equation (2.5).

In figure 7, we show two potential observables, the variance and the second moment of the distribution. In a community with two types, *S* = 2, both observables reflect the differences in growth, *f*_*i*_, and death rates, *ϕ*_*i*_. Only some variances overlap for distinct rates. In this sense, the second moment might provide more information to discriminate them. A set of similar observables could allow us to characterize the rates of empirical communities.

## 3. Discussion

Understanding the drivers of communities is one of the main objectives of ecological research [27,32]. In this work, we have used a stochastic death–birth model with immigration to investigate the equilibrium distribution of communities. Comparing cases where changes only depend on the abundances to cases where types have different birth or death rates, we have identified conditions leading to a robust occurrence-abundance pattern—often reported empirically.

Our approach acknowledges the intrinsic density dependence of large and diverse communities—addressing questions directly linked to empirical observations. In contrast to studies emphasizing biotic interactions [13,19,21,22], our model is more related to studies that focus on the differential adaptation to the environment [18,20]. As some of these studies, our results highlight the central role of immigration and biodiversity in community ecology [20,33].

We tested the reliability of our approach by reproducing known results of neutral adaptation [16]. Namely, the mean frequency of a type equals its immigration and the occurrence frequency increases in an S-shaped manner with the mean frequency, figure 1. We also see a good agreement to non-neutral simulations in the high immigration regime (figure 4*a*)—which is the focus throughout. The neutral results already capture the important role of immigration [15] but discard the frequency dependent effects of other types—for which biodiversity might be important.

The match between community level patterns of neutral models and empirical data has been documented extensively [6,8–10]. Still, some empirical evidence is at odds with neutral theory [34,35]. The mismatch with evolutionary history—including phylogenetic trees [34,35], is one of them. It has been observed that mild differences in adaptation can lead to full agreement [36]—indicating the need to consider models with differential adaptation, even if this is only mild.

Here, we considered a general death–birth model where large immigration consistently led to a robust occurrence-abundance pattern. Interestingly, evidence suggests that large immigration might indeed be common in various environmental and host-associated microbiomes [10]. Other microbiomes that deviate from the occurrence-abundance pattern have small immigration [10]. Such seems to be the case in *Caenorhabditis elegans*, where active destruction of microbes during feeding results in reduced immigration to the gut microbiome [37].

A second observation is that with differential adaptation, biodiversity takes a central role. In contrast to the simplest community of two interacting types (figures 2 and 3), highly diverse communities promote an occurrence-abundance pattern that resembles the neutral case (figure 5). With larger biodiversity, less extreme occurrences and mean frequencies are observed (compare figures 2 and 3 to figure 5). Our results agree with research showing that in the limit of high biodiversity, various neutral and non-neutral patterns converge at the community level [20].

Previous research has speculated about the ecological role of types based on their location in the occurrence-abundance curve [10]—the motivation being the possibility of identifying microbial taxa actively involved in biotic interactions. Our results indicate that such a direct identification of special taxa from occurrence-abundance curves remains challenging, mainly because neutral and non-neutral types can overlap (figure 6). We propose a way forward, based on the inclusion of new observables computed from data [30] (figure 7) combined with robust fitting approaches [31], on the line of Harris *et al.* [15].

Our focus at the level of types revealed the difficulty of assessing niche structure and neutrality from empirical data. While niche and neutral patterns can be indistinguishable at the community level, at the level of types, big differences are observed (figures 5 and 6). In microbial ecology, models have been commonly tested at the community level, where, embraced by a principle of parsimony, neutral interpretations have been suggested [8–10]. Our model suggests that this is indeed sensible for community level questions. However, for questions at the level of types—including that of ecological roles—general models including differential adaptation can not be avoided. In this case, no parsimonious preference can be given to neutral hypotheses.

The last observation calls for a broader discussion on terminology. As defined by Fisher & Mehta, a community is ‘statistically neutral’ if its distribution can not be distinguished from a distribution constructed under the assumption of ecological neutrality. However, ecological neutrality implies statistical neutrality, but statistical neutrality does not necessarily imply ecological neutrality [33]. As our results indicate, a reference to large immigration and biodiversity, rather than neutrality, is more accurate and prevents misleading interpretations, that in their worst form, could lead to unfounded generalizations or hold research questions back. On the contrary, our results suggest that numerous questions about neutrality, adaptation, and ecological roles, in microbial ecology and elsewhere are yet to be answered.

Although we mainly focused on microbial communities, our work can be framed in the larger macro-ecological literature. There, a substantial number of models have linked neutral and niche theories [13,18–22]. Heated debates have occurred; however, they have benefited from a close revision of the assumptions on the models and a careful discussion of their implications [6,20,36]. The observation of asymptotically equivalent patterns for neutral and non-neutral rates is one of their main results [20]. We believe microbial research can be guided along this line while offering powerful methods to investigate general ecological questions [15,30]. In particular, this is driven by the possibility to work, *in vivo* and *in vitro*, with large and diverse communities in much shorter time scales [38].

Finally, we mention some limitations of our work. A limitation of origin is that we considered a differential adaptation to the environment as the sole source of non-neutrality. Certainly, this is not true in nature, where types take part in numerous symbiotic interactions [13]. Therefore, any empirical application of our model should be preceded by evidence of little to no symbiosis. In addition, our model could be extended to include a dynamic external metacommunity and differential immigration to the local communities, similarly to the neutral approach of Harris *et al.* [15]. Although we provided a focused analysis of the occurrence-abundance pattern at equilibrium, future work could study its dynamics [39] and derive exact equations for these and other observables [30]. In fact, it might be necessary to consider observables at the level of types to distinguish between processes leading to equivalent community patterns [15]. Finally, identifying neutral and non-neutral types remains an open problem. The development of methods for parameter inference from data, similarly to [15,30], is an important next step.

## 4. Conclusion

Here we presented a general death–birth model with immigration. Using a combination of equations and stochastic simulations, we analysed the equilibrium distribution of abundances for communities equally or differently adapted to the environment. We observe that the community pattern of occurrence-abundance, often reported empirically, is consistently observed in conditions of large immigration and high diversity, regardless of the adaptation to the environment. However, at the level of types, differences in adaptation still lead to large changes.

## Data accessibility

The data generated and analysed during the current study can be simulated from the Python code available via GitHub at https://doi.org/10.5281/zenodo.5727664.
